# Cardiomyocyte-Specific Wt1 Is Involved in Cardiac Metabolism and Response to Damage

**DOI:** 10.3390/jcdd10050211

**Published:** 2023-05-12

**Authors:** Sandra Díaz del Moral, Maha Benaouicha, Cristina Villa del Campo, Miguel Torres, Nicole Wagner, Kay-Dietrich Wagner, Ramón Muñoz-Chápuli, Rita Carmona

**Affiliations:** 1 Department of Animal Biology, Faculty of Science, University of Málaga, 29071 Málaga, Spain; sandradiaz@uma.es (S.D.d.M.); chapuli@uma.es (R.M.-C.); 2 Department of Cell Biology, Genetics and Physiology, Faculty of Science, University of Málaga, 29071 Málaga, Spain; benaouicha.maha@gmail.com; 3 Cardiovascular Development Program, Centro Nacional de Investigaciones Cardiovasculares, CNIC, 28029 Madrid, Spain; cristina.villa@cnic.es (C.V.d.C.); mtorres@cnic.es (M.T.); 4 Centro de Investigación Biomédica en Red de Enfermedades Cardiovasculares (CIBERCV), 28029 Madrid, Spain; 5 Université Côte d’Azur, CNRS, INSERM, iBV, 06108 Nice, France; nwagner@unice.fr (N.W.); kwagner@unice.fr (K.-D.W.); 6 Department of Human Anatomy, Legal Medicine and History of Science, Faculty of Medicine, University of Málaga, 29071 Málaga, Spain

**Keywords:** Wilms’ tumor suppressor gene, cardiomyocytes, cardiac metabolism

## Abstract

The Wilms tumor suppressor gene (Wt1) encodes a C2H2-type zinc-finger transcription factor that participates in transcriptional regulation, RNA metabolism, and protein–protein interactions. WT1 is involved in the development of several organs, including the kidneys and gonads, heart, spleen, adrenal glands, liver, diaphragm, and neuronal system. We previously provided evidence of transient WT1 expression in about 25% of cardiomyocytes of mouse embryos. Conditional deletion of Wt1 in the cardiac troponin T lineage caused abnormal cardiac development. A low expression of WT1 has also been reported in adult cardiomyocytes. Therefore, we aimed to explore its function in cardiac homeostasis and in the response to pharmacologically induced damage. Silencing of Wt1 in cultured neonatal murine cardiomyocytes provoked alterations in mitochondrial membrane potential and changes in the expression of genes related to calcium homeostasis. Ablation of WT1 in adult cardiomyocytes by crossing αMHC^MerCreMer^ mice with homozygous WT1-floxed mice induced hypertrophy, interstitial fibrosis, altered metabolism, and mitochondrial dysfunction. In addition, conditional deletion of WT1 in adult cardiomyocytes increased doxorubicin-induced damage. These findings suggest a novel role of WT1 in myocardial physiology and protection against damage.

## 1. Introduction

The Wilms’ tumor suppressor gene (*Wt1*) encodes a C2H2-type zinc-finger transcription factor that participates in transcriptional regulation, RNA metabolism, and protein–protein interactions. WT1 is critically involved in the development of various organs, including the kidneys, gonads, spleen, adrenal glands, liver, and diaphragm [1]. WT1 is highly expressed in the embryonic epicardium, where it regulates a process of epicardial–mesenchymal transformation and the differentiation of epicardial-derived cells. These mesenchymal cells give rise to fibroblasts, endothelial cells, and smooth muscle from the cardiac connective and vascular tissues [2,3,4].

We recently provided evidence of WT1 expression in a subpopulation of cardiomyocytes of mouse embryos and adults [5,6]. Conditional deletion of Wt1 in the cardiac troponin T lineage caused abnormal sinus venosus and atrium development, a thin ventricular myocardium, and, in some cases, interventricular septum and cardiac wall defects [5]. Thus, myocardial WT1 expression is required for normal cardiac development. However, the role of WT1 in adult cardiomyocytes has not yet been addressed. WT1 is normally expressed at low levels in these cells, and a significant upregulation has been reported after myocardial infarction [6]. Furthermore, the systemic deletion of this gene in adult mice provokes multiple organ failure, including significant changes in cardiac size [7]. Other postnatal roles of this transcription factor have been described in recent years. For example, we have shown that re-expression of WT1 in stellate cells is necessary for pancreas repair after damage [8]. WT1 also plays a role in modulating fibrogenesis in hepatic stellate cells after injury [9]. Finally, WT1 is also temporally upregulated in the epicardium and in the endothelial cells of the myocardial infarcted area and border zone [10,11,12]. Thus, further characterization of the emerging potential roles played by WT1 in adult organs is warranted.

We aimed to explore potential functions of WT1 in postnatal cardiomyocytes. For this purpose, we employed the deletion of WT1 in postnatal cardiomyocytes in vitro and in vivo, analyzing consequences both in physiological conditions as well as in the response to pharmacologically induced damage.

## 2. Materials and Methods

### 2.1. Animal Models

The animals used in our research program were handled in compliance with the institutional and European Union guidelines (Directive 2010/63/EU of the European Parliament) for animal care and welfare. The procedures used in this study were approved by the Committee on Ethics of Animal Experiments of the University of Málaga (procedure code 2018-0018). According to these procedures, the mice were euthanized via cervical dislocation.

For conditional deletion of WT1 in cardiomyocytes, we generated tamoxifen-inducible WT1 mutants by crossing αMHC^merCremer^ mice [13], directing expression of a tamoxifen-inducible Cre recombinase (MerCreMer) to juvenile and adult cardiac myocytes, with homozygous WT1-floxed mice, where the first exon of *Wt1* is flanked by loxP sites [3]. To induce recombination in αMHC^merCremer^; WT1^loxP/loxP^ mice, they were fed for 1 month with pellets containing tamoxifen citrate at a concentration of 400 mg/kg (Teklad, TD.55125, Indianapolis, IN, USA) [14]. Then, the animals were fed again with a normal diet for a minimum period of two weeks before starting the experiments, to eliminate possible acute side effects of tamoxifen in the results. As controls, we used both mice expressing Cre recombinase with normal *Wt1* alleles and mice lacking Cre expression with WT1^loxP/loxP^ mice. We did not observe differences between these controls. Control mice always received the same treatment as the mutant mice in each experiment.

The mice were euthanized at different intervals to investigate different aspects of the mutant phenotype. For acute DOX treatment, three controls and four mutants were injected, while nine controls and six mutants were used for chronic DOX treatment. The number of mice selected and examined in each procedure is detailed in the following paragraphs and in the results, and this information is also summarized in Supplementary Table S1.

### 2.2. Neonatal Cardiomyocyte Isolation and Culture

Neonatal cardiomyocytes were isolated following the procedure of Da Silva et al. [15]. Hearts from newborn mice (C57BL/6 strain, Janvier Labs, Le Genest-Saint-Isle, France) at postnatal day 2 (P2) were collected in high-glucose DMEM (Gibco, Courtaboeuf, France, 11965092) supplemented with 10% fetal calf serum (FCS) (Gibco), 1 mM sodium pyruvate (Gibco, 11360070), and 100 U/mL penicillin and 100 μg/mL streptomycin (Gibco, 15140122). Then, the hearts were minced and processed following 4 steps of digestion with 2.5% trypsin (T4799, Sigma, Saint-Quentin-Fallavier, France) in DMEM without FCS at 37 °C in a shaker at 200 rpm, for 15 min each time. After each incubation step, the supernatant was mixed with DMEM plus FCS to block trypsin activity. Once the digestion was finished, all the supernatant was collected and the cells were passed through a 70 µm filter (Miltenyi Biotec, Paris, France, 130-098-462), and then centrifuged for 5 min at 1600 rpm and washed with Dulbecco’s Phosphate Buffered Saline (DPBS) (Gibco, 14190144) plus 2% FCS and 0.5 mM of EDTA (Gibco, Courtaboeuf, France). The supernatant was removed, and an approximately final number of 10^7^ cells were resuspended in 90 μL of DPBS containing FCS and EDTA and labeled for 30 min with 10 μL of magnetic microbeads from the Neonatal Cardiomyocyte Isolation Cocktail (Miltenyi Biotec, 130-100-825) at 4 °C, which will bind to all cell types but cardiomyocytes. Cells were later negatively selected by passing them through an MS column (Miltenyi Biotec, 130-042-201), previously washed with 500 μL of DPBS with FCS and EDTA, and placed in a MiniMACS separator magnet (Miltenyi Biotec, 130-042-102). Cardiomyocytes were eluted through 3 washes with 500 μL of supplemented DPBS each, while the other cell types were retained in the column. After a 5 min centrifuge at 1600 rpm, the pellet was resuspended in 12 mL of high-glucose DMEM plus FCS, sodium pyruvate, and penicillin/streptomycin, and cardiomyocytes were plated on two 6-well plates pre-coated with 1% collagen type I (C3867, Sigma)) in DPBS for 1 h at 37 °C, in a final volume of 2 mL per well. Cardiomyocytes were maintained in a cell culture incubator at 37 °C (95% air, 5% CO_2_) in high-glucose DMEM with FCS, sodium pyruvate, and penicillin/streptomycin. The medium was changed twice a week until cells reached 80% confluency.

### 2.3. Wt1 Silencing via Lentiviral shRNA Particles in Cultured Cardiomyocytes

Lentiviral particles expressing Wt1 shRNA (short hairpin RNA), or NC-RNA (non-coding RNA) as a control, were co-transfected using the calcium phosphate method with Gag/pol+ and VSV g plasmids into 5 × 10^6^ HEK293 cells in DMEM with 10% FCS in 10 cm dishes. After 48 h at 37 °C in 5% CO_2_, the supernatant containing the viral particles was collected and filtered through a 0.45 mm mesh to infect neonatal cardiomyocytes after 24 h of incubation.

### 2.4. RNA Isolation, Reverse Transcription, and qRT-PCR of Neonatal Cardiomyocytes

Total RNA was isolated from seven samples of shRNA and seven samples of NC-RNA cultured cardiomyocytes using TRIzol reagent (Thermo Scientific, Courtaboeuf, France, 15596026) according to the manufacturer’s protocol. The samples were pools of cardiomyocytes obtained from four independent cultures for each condition. The RNA was then quantified using a NanoDrop 2000/2000c Spectrophotometer (Thermo Scientific), and the reverse transcription was performed with the Thermo Scientific Maxima First Strand cDNA Synthesis Kit (Thermo Scientific, K1672) to achieve first-strand cDNA synthesis. The final volume of cDNA was diluted ten times in nuclease-free water, and 2.5 ng of each sample was used to perform the quantitative real-time PCR (qRT-PCR) with the PowerUp SYBR Green Master Mix (Thermo Scientific, A25742) using the StepOne Plus thermocycler (Thermo Scientific). The expression of the housekeeping genes Gapdh, Rplp0, and β-actin was measured for each sample, and the expression values for all samples, which were loaded in triplicate, were calculated using the ΔCt method, after subtracting the mean value of the housekeeping gene Ct from the Ct of the different genes of interest [16]. Normalization of each sample against the mean value of the NC-RNA samples was performed to obtain the relative gene expression values. Sequences of the primers used for qPCR are provided in Supplementary Table S2.

### 2.5. Protein Extraction, Quantification, and Western Blot

Neonatal cardiomyocytes were incubated with 75 μL of RIPA buffer containing protease inhibitors, maintained on ice for 30 min, and kept overnight at 4 °C in a shaker. The day after, the samples were centrifuged at 4 °C for 30 min at 13,000 rpm, and then the supernatant containing the proteins was taken into a new tube and stored at −80 °C. The quantification method was performed using a colorimetric BCA assay (Uptima, Interchim, Montluçon, France) in transparent 96-well plates. After preparing a BSA standard curve that ranged from 0 to 1 mg/mL, 5 μL of each sample and each standard value were loaded in triplicate. The samples were first diluted 5 times in distilled water. The plate was then incubated in the darkness for 30 min with 80 μL of BCA reagent per well, and the quantification was obtained via measuring the absorbance in a plate spectrophotometer (Biorad, Duesseldorf, Germany) at 560 nm.

Once the samples were quantified, 30 μg of protein was prepared in Laemmli buffer and denatured for 5 min at 95 °C. A final volume of 90 μL per sample was loaded on 10% acrylamide gel (acrylamide–bisacrylamide 37.5/1). After 45 min of electrophoresis, the proteins were transferred to PVDF membranes (Biorad, 162-0177) that had previously been activated in methanol for 40 s. The membranes were blocked for 1 h in the shaker with 5% milk powder (Difco, 232100) prepared in PBS plus 0.05% of Tween 20 (Sigma, P9416), and then incubated overnight with gentle shaking at 4 °C with the rabbit anti-Wt1 primary antibody (Abcam, Cambridge, UK, Ab89901) diluted at 1:1000 in 2.5% milk prepared in PBS plus 0.05% of Tween 20. The next day, the membranes were washed and incubated for 1 h in the shaker at room temperature with the anti-rabbit peroxidase-labeled secondary antibody (Vector Laboratories) diluted at 1:2000 in 2.5% milk dissolved in PBS with 0.05% of Tween 20. After a 5 min incubation step with the enzyme-specific substrate, ECL Select Western blotting detection reagent (GE Healthcare, Buc, France, RPN2235), the chemiluminescent signal was detected with the ImageQuant LAS 600 (GE Healthcare). After this, the membrane was incubated for 15 min in the shaker with 10 mL of stripping buffer (Gene Bio-Application Ltd., Yavne, Israel, ST010) to remove the Wt1 binding, washed 5 times with distilled water for 5 min each, and blocked again with milk for 1 h in the shaker. The membrane was incubated overnight with the rabbit monoclonal anti-Gapdh antibody (Abcam, ab181602), and the day after, the signal of the housekeeping protein, Gapdh, was detected after a 1 h incubation step with the same secondary antibody and detection reagent.

Details of the antibodies used for the Western blot are provided in Supplementary Table S3.

### 2.6. Flow Cytometry

Cultured neonatal cardiomyocytes were detached after 10 min of enzymatic digestion with Trypsin-EDTA (Gibco, 25300-054) at 37 °C. Three wells were used per condition, with a final number of three samples of NC-RNA cells and three samples of shRNA cardiomyocytes. The cell suspensions were centrifuged and washed using 2% FCS and 0.5 mM EDTA in PBS. For intracellular calcium measurements, the cells were incubated with 10 µM Fluo-4 AM (Biotium, Fremont, CA, USA, 50018) in 2% FCS and 0.5 mM EDTA in PBS at room temperature for 15 min. For mitochondrial analysis, the cell suspensions were stained with 100 nM of deep red MitoTracker (Thermo Scientific, M22426) in PBS containing 2% FCS and 0.5 mM EDTA for 30 min at 37 °C, in the darkness, to detect possible differences in their mitochondrial content. To compare the mitochondrial membrane potential of each condition, 100 nM PE TMRE (tetramethylrhodamine ethyl ester perchlorate) (Sigma, 87917) was prepared in 2% FCS and 0.5 mM EDTA in PBS, and the samples were stained for 30 min in the darkness at 37 °C. A non-stained negative control, composed by a pool of all the samples, was prepared for each experiment to remove the non-specific signal in the analysis. To determine possible changes in the percentage of cells undergoing apoptosis, the PE Annexin V Apoptosis Detection Kit I (BD Pharmingen, San Diego, CA, USA, 559763) was used. The cell suspensions were incubated with 5 µL of PE Annexin V and 5 µL of 7-AAD (7-Amino-Actinomycin) in 1X binding buffer, as contained in the kit, for 15 min in the dark. This analysis was carried out using two different controls besides the non-stained negative control: one containing just 7-AAD, and another one with just PE Annexin. Each experiment was performed using a CytoFLEX LX cytometer (Beckman Coulter, Brea, CA, USA), and the obtained data were then analyzed with the CytExpert software (Beckman Coulter).

For flow cytometry analysis of adult mice, dissected hearts were minced into small fragments, washed in PBS, and dissociated in a 0.5 mg/mL Liberase TM solution in PBS (Sigma, 5401127001) at 37 °C for 7 min in agitation. The digestion was stopped with four volumes of cold 10% fetal bovine serum in PBS. The dissociated cells were centrifuged, resuspended in cytometry buffer (PBS plus 2% fetal bovine serum and 10 mM HEPES), and filtered through a 70 μm nylon mesh. Then, cells were incubated on ice in the dark with the fluorochrome-conjugated antibodies and DAPI (Sigma, D9542).

Cardiomyocytes were identified using a mitochondrial staining reagent (MitoTracker Deep Red, Thermo Scientific, M22426) [17]. We incubated the cell suspension, previously stained with anti-CD31, in the MitoTracker solution (100 nM in cytometry buffer) at 37 °C for 30 min. Another mitochondrial marker, TMRE, was also used to assess changes in the mitochondrial membrane potential [18]. Cardiac cell suspensions previously stained with anti-CD31 were incubated with TMRE (100 nM in cytometry buffer) for 30 min at 37 °C in the dark before analysis.

Analytical flow cytometry was performed in an FACS Verse flow cytometer. Data were analyzed with Kaluza Analysis software (version 2.1) and are displayed in tables as mean ± standard error of mean. Negative controls (Cre-negative littermates) and isotypic antibodies allowed setting of the gates.

Details of the antibodies used for flow cytometry are provided in Supplementary Table S3.

### 2.7. Proliferation Assay in Neonatal Cardiomyocytes

To measure cell proliferation in both conditions, a colorimetric immunoassay was performed on three controls and three mutants based on the incorporation of BrdU (5-bromo-29-deoxyuridine) in DNA synthesis, using the Cell Proliferation ELISA BrdU kit (Roche, Zürich, Switzerland, 11 647 229 001) according to the manufacturer’s instructions. Cells were first obtained through a digestion step with trypsin and then plated in a 96-well ELISA microplate with DMEM. After that, the samples were incubated with 10 µM BrdU for 5 h. The final colorimetric results were obtained via measuring the absorbance with the iMark microplate absorbance reader (Bio-Rad) at 450 nm.

### 2.8. Cell Viability Assay in Cultured Neonatal Cardiomyocytes

The RealTime-Glo MT Cell Viability Assay (Promega, Madison, WI, USA, G9711) was performed in cultured neonatal cardiomyocytes, using three NC-RNA and three shRNA cell samples, to evaluate possible differences between the two conditions. Cells were collected through a 5 min incubation procedure with Trypsin-EDTA and then counted using the Beckman Z1 coulter counter (Beckman Coulter, Brea, CA, USA). A final number of 2000 cells of each condition were incubated for 30 min at 37 °C with 20 µL of RealTime-Glo reagent in DMEM medium. Luminescence was measured using a Modulus Luminometer (Promega, Madison, WI, USA). For this study, the cell viability assay was first measured in cultured cardiomyocytes that were not infected with lentiviral particles as a control to confirm that the transduction did not affect viability.

### 2.9. Laminin Immunostaining and Confocal Microscopy

Ventricular fragments obtained via dissection of the hearts were fixed in 4% paraformaldehyde for 4–6 h, dehydrated, and paraffin-embedded. Deparaffinized sections were washed in Tris-PBS (TPBS) and blocked for non-specific binding with SBT (16% sheep serum, 1% bovine albumin, 0.1% Triton X-100 in TPBS). Single immunofluorescence was performed via incubating the sections overnight at 4 °C with a rabbit polyclonal anti-laminin antibody (Sigma, L9393) diluted 1:25 in TPBS, washing in TPBS, and incubating with the corresponding fluorochrome-conjugated secondary antibody for 1 h at room temperature. Nuclei were counterstained with DAPI (Sigma, D9542). Sections were mounted in PBS:Glycerol 1:1.

### 2.10. Doxorubicin Treatment

To assess the consequences of a doxorubicin treatment, nine control and six mutant mice were injected intraperitoneally (i.p.) once per week with doxorubicin (5 mg/kg) for four weeks at a final dose of 20 mg/kg [19]. Electrocardiographic studies were carried out three and six months after the final injection and mice were euthanized six months after the end of the treatment. In some experiments, as indicated in the results, an acute doxorubicin treatment was applied to three control and four mutant mice via a single intraperitoneal injection of 20 mg/kg [20] one month after conditional WT1 deletion. These mice were euthanized three days after the injection. Doxorubicin treatment did not affect the survival of the mice.

### 2.11. Electrocardiography (ECG)

The anesthesia used to perform the ECGs was ketamine (64 μg/g body weight)/xylazine (10 μg/g) in PBS (i.p.). After 15 min, electrocardiograms were obtained from mice using BioAmp PowerLab 8/35 (AD Instruments, Dunedin, New Zealand) for the data acquisition. The negative electrode and positive electrode were placed in the upper right and lower left limbs, respectively. The neutral pole was placed in the lower right limb. We analyzed a section of the ECG for 1 min and we used the ECG module of the LabChart 8.0 software and the preset mouse settings. The number of analyzed mice is detailed in Tables 1 and 3.

### 2.12. Proteomics

Proteomic analysis was performed on ventricular samples of four control mice and three mice with conditional deletion of WT1 in cardiomyocytes. Hearts of mice treated chronically with doxorubicin were also analyzed (two controls and three mutants). The protein concentrations of ventricular samples were determined by the bicinchoninic acid protein assay and adjusted to the same concentration (1μg/uL). For reduction and alkylation, 5 μL of 200 mM Tris(2-carboxyethyl) phosphine was added and incubated at 55 °C for 1 h. The proteins were alkylated with 60 mM iodoacetamide at room temperature for 30 min and protected from light. Samples were subjected to acetone precipitation to purify proteins via incubation with 6 volumes of ice-cold acetone at −20 °C for 4 h. Precipitated proteins were centrifuged at 8000× *g* for 10 min at 4 °C and the pellet redissolved in 100 μL of 50 mM triethylammonium bicarbonate buffer (pH 8.5). Proteins were digested by trypsin (Pierce trypsin protease, MS grade) at a ratio of 1:50 (trypsin/protein, *w*/*w*) by incubating overnight at 37 °C.

For quantitative proteomics, each digested protein sample was labeled with an appropriate TMT2plex Isobaric Label Reagent (Thermo Scientific, Waltham, MA, USA) according to the protocol supplied by the manufacturer. The wild-type and knockout peptide samples were labeled with reagents TMT-126 and TMT-127, respectively. To quench the TMT reaction, 8 μL of 5% hydroxylamine was added to each sample and incubated for 15 min at room temperature. All labeled peptides were combined in equal amounts and dried using a speed vacuum system.

The peptides were dissolved in 0.1% formic acid and analyzed using a Q-Exactive HF-X Hybrid Quadrupole-Orbitrap Mass Spectrometer (Thermo Fisher Scientific) connected to an Easy-nLC 1200 UHPLC (Thermo Fisher Scientific). Solvent A and solvent B were 0.1% formic acid in water and 0.1% formic acid in 80% acetonitrile, respectively. Peptide separation was performed using a trap column (Acclaim PepMap 100, 75 μm × 2 cm, C18, 3 μm, 100 A, Thermo Fisher Scientific) at a flow rate of 20 μL/min and eluted onto a 50 cm analytical column (PepMap RSLC C18, 2 um, 100 A, 75 μm × 50 cm, ThermoFisher Scientific) with a linear gradient of 2% to 20% solvent B (0.1% formic acid in 80% acetonitrile) for 240 min, followed by a 30 min gradient from 20% to 35% solvent B and finally, to 95% solvent B for 15 min before re-equilibration to 2% solvent B at a constant flow rate of 300 nL/min. The peptides were positively ionized using a nanospray ion source and mass spectrometry was conducted in top 20 data-dependent mode with the following settings: 2.2 kV ion spray voltage; 320 °C ion capillary temperature; 350–1500 mass scan range for full MS and 350–1500 scan range for MS/MS; MS resolution 120,000 for full MS and 30,000 for data-dependent MS/MS; collision energy 32 with high-energy collisional dissociation mode; MS/MS isolation window 0.7 m/z; and dynamic exclusion 20 s.

The peptides and proteins were identified and quantified using Proteome Discoverer^TM^ 2.4 (Thermo Fisher Scientific) platform and SwissProt *Mus musculus* protein database version 2017.10.25 (25,097 sequences). The search parameters were set as follows: two missed tryptic cleavage sites were allowed; full MS and MS/MS tolerances were 10 ppm and 0.02 Da, respectively; fixed modification of carbamidomethylation on Cys, variable modification of oxidation on Met, and *N*-terminal acetylation on protein were specified; proteins required a minimum of two peptides. Peptide spectral matches and consecutive protein assignments were validated using the Percolator^®^ algorithm by imposing a strict cut-off of 1% false discovery rate (FDR). To calculate the ratio of TMT-labeled proteins between the wild type and knockout, abundances were based on precursor intensities. Normalization was performed based on total peptide amount, and samples were scaled based on the average (for every protein and peptide the average of all samples is 100). Protein ratios were directly calculated from the grouped protein abundances. Abundance ratio *p*-values were calculated using ANOVA based on the abundance of individual proteins.

### 2.13. Fibrosis and Image Analysis

The degree of fibrosis was assessed in histological sections stained with picrosirius red staining [21]. The analysis was performed on ventricular sections of two control mice and three mice with conditional deletion of WT1 (two months after deletion, 21 and 27 sections analyzed, respectively). Two control mice and two mutant mice were also analyzed six months after the deletion, using 19 and 13 sections, respectively. Hearts of mice treated with doxorubicin were also analyzed. One month after the treatment, 37 and 36 sections from three controls and four mutant mice, respectively, were analyzed. Six months after the treatment, 12 and 15 sections were analyzed from three controls and three mutants, respectively. The size of cardiomyocytes was assessed in histological sections immunostained with anti-laminin antibody as described above. In both cases, stained areas were quantified on digitalized images using ImageJ software.

### 2.14. Statistics

Data shown are given as means ± SD of the number of independent experiments indicated. Statistical comparison was performed using two-tailed Student’s t-test or one-way ANOVA. We used the Z-test when we compared percentage scores relative to controls (expression and flow cytometry data). *p* < 0.05 was considered as statistically significant.

## 3. Results

### 3.1. Silencing of Wt1 in Cultured Neonatal Cardiomyocytes Induces Changes in Mitochondrial Membrane Potential

**Figure 1:**
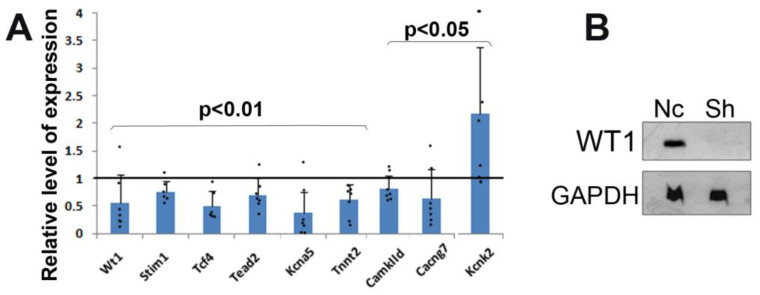
Changes in the expression levels of different genes after silencing Wt1 through lentiviral shRNA transduction in cultured mouse cardiomyocytes. Control cardiomyocytes were transduced with non-coding RNA. N = seven mutant and seven control samples (pools of cardiomyocytes from four independent cultures for each condition). (**A**) The mean level of expression ± S.D. assessed through qPCR is shown relative to the mean level of the control samples, represented by the horizontal line. *Stim1*, Tcf4, Tead2, CamkIId, Cacng7, Kcna5, and Tnnt2 were significantly downregulated, while the expression of Kcnk2 was significantly upregulated (Z-test). (**B**) Western blot of WT1 in cardiomyocytes transfected with non-coding (Nc) and shRNA. Black dots represent individual values.

WT1 expression was significantly downregulated in neonatal mouse cardiomyocytes in primary cultures through shRNA transduction. The expression of WT1 was reduced by 50.3 ± 18.0% as shown through qRT-PCR (Figure 1A, *p* < 0.01, Z-test). WT1 protein expression was also strongly reduced in mutant cardiomyocytes (Figure 1B).

We analyzed through quantitative RT-PCR a set of genes involved in cardiac physiology. Interestingly, we found significant downregulation of several genes in neonatal cardiomyocytes with reduced WT1 expression, namely Stim1, Tcf4, Tead2, Kcna5, Tnnt2, CamkIId, and Cacng7. The expression of Kcnk2 was significantly upregulated. (Figure 1).

**Figure 2:**
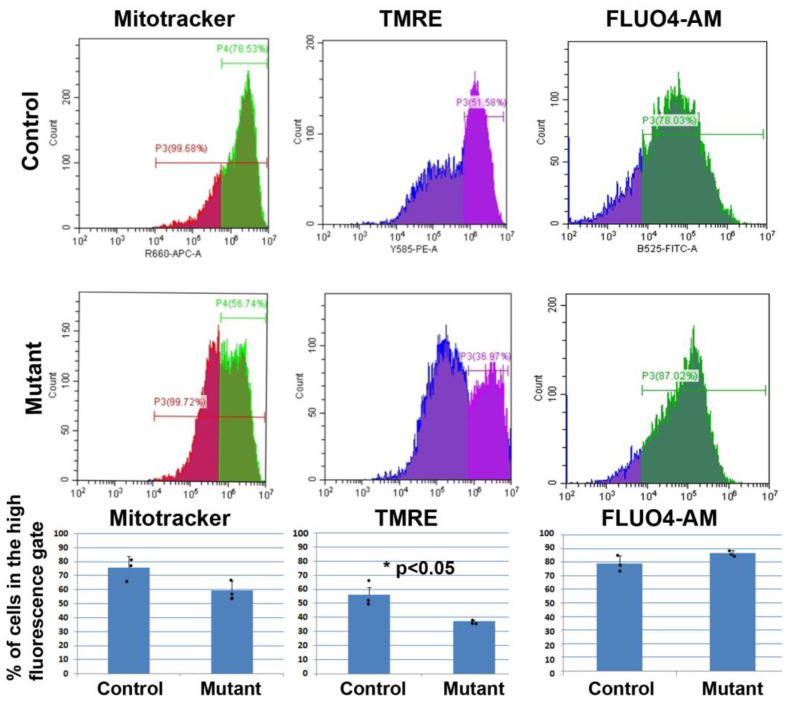
Representative cytograms of control and mutant (with downregulation of WT1) cardiomyocytes. The significant decrease in TMRE fluorescence suggests mitochondrial dysfunction. The mitochondrial load, estimated by MitoTracker staining, and intracellular calcium levels were statistically not significant (*p* < 0.06 and 0.08, respectively. n = three mutant and n = three control samples; error bars represent standard deviations, one-way ANOVA test). * *p* < 0.05.

The estimation of the mitochondrial load through MitoTracker staining revealed a potential decrease in WT1-deficient cardiomyocytes close to the level of statistical significance (*p* < 0.06, Student’s test, n = three WT1 shRNA and n = three nc shRNA samples each) (Figure 2). Furthermore, TMRE staining revealed a significant decrease in the level of the mitochondrial membrane potential of the WT1-deficient cardiomyocytes (*p* < 0.05, one-way ANOVA, n = three WT1 shRNA and n = three nc shRNA samples each) (Figure 2).

The levels of resting intracellular Ca^2+^ were estimated via Fluo-4 AM staining and flow cytometry. Mutant cardiomyocytes showed a 10% increase in intracellular calcium levels, albeit this was statistically not significant (*p* < 0.08, n = three WT1 shRNA and n = three nc shRNA samples) (Figure 2).

### 3.2. Conditional Deletion of WT1 in Adult Cardiomyocytes (αMHC^merCremer^;WT1^loxP/loxP^) Induces Fibrosis and Hypertrophy

**Figure 3:**
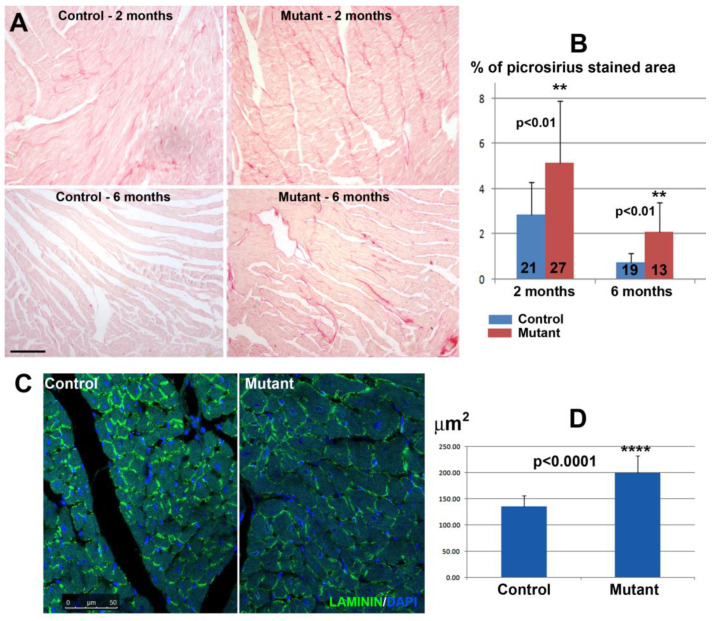
Mutant mice with conditional deletion of WT1 in cardiomyocytes showed a significant increase in interstitial fibrosis and cardiomyocyte size as compared to control mice. (**A**) The upper images show representative picrosirius staining of controls and mutants two months after deletion of WT1. Fibrosis decreased after six months but the difference between mutant and control persisted, since the fibrosis was almost resolved in the control mice while it remained relatively high in the mutants (scale = 200 µm). (**B**) The graph shows the percentage of the surface stained with picrosirius red (n = two controls and n = three mutants two months after WT1 deletion, n = two controls, and n = two mutants six months after WT1 deletion, respectively. The number of sections studied is shown on the columns). (**C**) Representative images of ventricular myocardium sections immunostained with laminin of control and mutant mice two months after WT1 deletion (scale = 50 µm). (**D**) The graph shows the mean transversal area of the cardiomyocytes in seven sections of two mutant and eleven sections of three control mice (552 and 678 measurements, respectively) two months after the deletion (means ± SD, Student’s *t*-test). ** *p* < 0.01, **** *p* < 0.0001.

To explore the potential functional role of WT1 in adult cardiomyocytes, we deleted WT1 in the myocyte population through tamoxifen administration and analyzed the hearts one, two, or six months after tamoxifen treatment. We found an increase in cardiac fibrosis two and six months after cardiomyocyte-specific WT1 deletion. Fibrosis was evidenced by the area covered by collagen fibers stained with picrosirius red (Figure 3). Six months after the deletion, fibrosis decreased but the relative difference between control and mutant hearts increased due to the faster resolution of fibrosis in the control. Control mice treated with tamoxifen showed some increase in the degree of fibrosis as compared with wild-type mice, but this increase was statistically not significant (data not shown). The mean cross-sectional area of cardiomyocytes was significantly higher in mutant mice two months after WT1 deletion (199.2 ± 12.7 μm^2^ vs. 135.9 ± 6.1 μm^2^ in control mice) compared to controls, indicating cardiomyocyte hypertrophy (Figure 3). However, we did not observe significant differences in the heart weight/body weight ratio of mutant and control mice (0.96% ± 0.28 in control, n = 6; 0.98% ± 0.26 in mutants, n = 6).

### 3.3. Conditional Deletion of WT1 in Adult Cardiomyocytes Induces Mitochondrial Dysfunction

**Figure 4:**
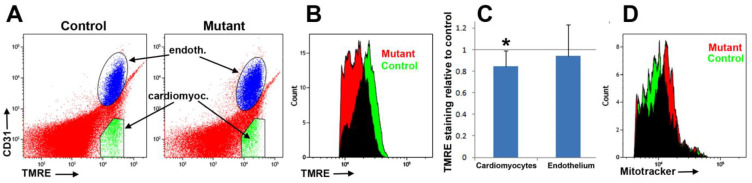
(**A**) Representative cytograms of cardiac cells (from six control and seven mutant mice) stained with anti-CD31 and TMRE. Gate green represents the cardiomyocyte population (CD31^-^/TMRE^high^). (**B**) Representative histograms of the TMRE signal in a mutant (red) and a control (green) population of CD31^-^/TMRE^high^ cells (cardiomyocytes). (**C**) Quantification of TMRE staining in the mutant mice relative to control staining. TMRE signal in mutant cardiomyocytes represents 84.5% of the mean control signal (*p* < 0.05, Z-test). However, endothelial cells from the mutant mice did not show significant differences relative to controls. (**D**) Representative histograms of the MitoTracker signal in a mutant (red) and a control (green) population of MitoTracker^high^/CD31- cells (cardiomyocytes). The difference in the mitochondrial load was not significant. * *p* < 0.05.

Our data obtained in neonatal cardiomyocytes suggest a potential role of WT1 in regulating mitochondrial function. Therefore, we sought to explore whether this was also the case in adult cardiomyocytes. The WT1-deficient cardiomyocyte population showed a lower signal of TMRE staining two months after tamoxifen administration. This signal was 84.5 ± 5.9% relative to that found in control cardiomyocytes (seven mutant and six control mice) (*p* < 0.05, Z-test). This observation suggests a lower mitochondrial membrane potential. Endothelial cells used as a control showed similar median values in control and mutant mice (Figure 4). Mitochondrial load did not show significant differences between control and mutant mice (Figure 4B).

### 3.4. Conditional Deletion of WT1 in Adult Cardiomyocytes Did Not Substantially Alter Electrocardiogram Parameters

We addressed using ECG measurements potential global changes in cardiac electrophysiological function 15 days after tamoxifen administration. ECG parameters showed no substantial changes after cardiomyocyte-specific deletion of WT1. Only the QRS complex was significantly shorter in mutant mice (13.6 ms vs. 14.6 ms, *p* < 0.05, Student’s *t*-test) (Table 1).

### 3.5. Proteomic Analysis Shows Alterations in Cardiomyocyte Metabolism after Conditional Deletion of Wt1

To further characterize the cellular phenotype of WT1-deficient cardiomyocytes, we performed proteomics assays one month after tamoxifen administration. The results were analyzed following two different approaches using the WebGestalt platform (http://www.webgestalt.org/ accessed on 21 April 2023). On one hand, over-representation analysis (ORA) was performed on a list of 130 proteins (17 downregulated, 113 upregulated) with an abundance ratio <0.9 or >1.1 and a *p*-value < 0.05. On the other hand, the whole list of 826 proteins with a high protein FDR confidence level and a minimum of two peptides identified was analyzed with a gene set enrichment analysis method (GSEA).

According to ORA analysis (using the KEGG pathway database), biological processes significantly over-represented in the 130 proteins selected mainly included metabolic pathways, including amino acid degradation, glutathione metabolism, and oxidative phosphorylation. PPAR (peroxisome-proliferator-activated receptor) signaling and the cardiac contraction pathways were also altered by myocardial WT1 deletion (Table 2). The phenotypes related to the differentially expressed proteins mainly included abnormal fatty acid metabolism and abnormal heartbeat. GSEA analysis of the full set of proteins showed a significant downregulation of pathways related to the electron transport chain and oxidative phosphorylation.

Supplementary Table S4 shows 13 proteins differentially expressed in the heart of mutant and control mice with an adjusted *p*-value of <0.05, 3 of which were downregulated and 10 of which were upregulated in the mutant. The altered glutathione metabolism suggested by ORA analysis is represented here by the upregulation of two glutathione S-transferases. 3-methylcrotonyl-CoA carboxylase catalyzes a critical step for leucine and isovaleric acid catabolism, and it appears strongly upregulated in mutant mice. Thus, proteomic analysis of the WT1 conditional ablation in cardiomyocytes suggests a strong impact on the metabolism of these cells, with a lower production of energy and alterations in fatty acid metabolism.

### 3.6. Conditional Deletion of WT1 in Adult Cardiomyocytes Increases the Damage Induced by Doxorubicin Treatment

**Figure 5:**
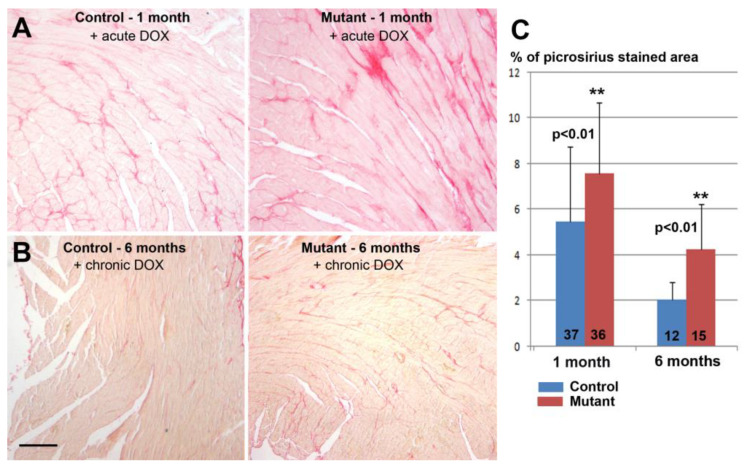
Mutant mice with conditional deletion of WT1 in adult cardiomyocytes and treated with doxorubicin showed a significant increase in interstitial fibrosis in the hearts as compared to control mice. (**A**) Representative picrosirius staining one month after the deletion of WT1 and three days after a single-dose doxorubicin (DOX) treatment. (**B**) Fibrosis decreased after six months of Wt1 deletion and chronic administration of doxorubicin, but the difference between mutant and control persisted. Note the fastest regression of fibrosis was observed in the control mice (scale = 200 µm). (**C**) The graph shows the percentage of the surface stained with picrosirius red (means ± SD, n = three control mice and n = four mutant mice with acute DOX treatment, n = three control mice and n = three mutant mice with chronic DOX treatment, respectively. The number of sections studied is shown on the columns). Student’s *t*-test, ** *p* < 0.01.

Doxorubicin treatment did not affect the survival of either control or mutant mice. Mutant hearts from mice treated with doxorubicin showed significantly increased fibrosis as compared with control mice, either in acute or chronic treatments (Figure 5).

**Figure 6:**
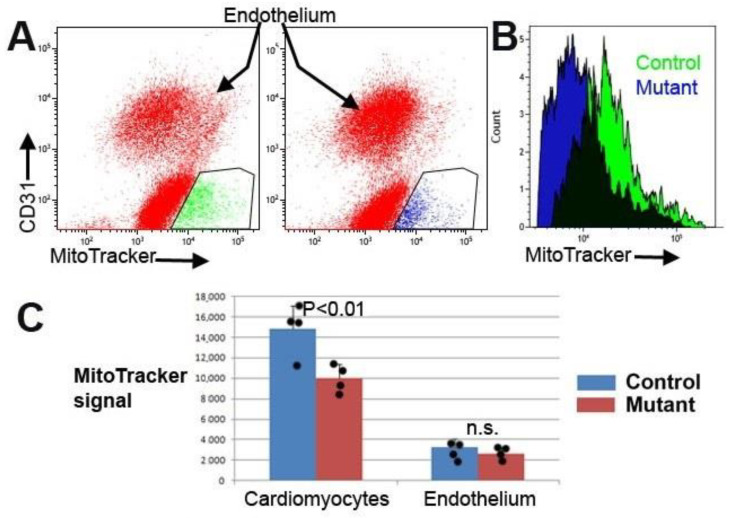
Conditional deletion of WT1 provoked a significant reduction in the mitochondrial load of cardiomyocytes after acute doxorubicin treatment. (**A**) Representative cytograms of cardiac cells from control and mutant hearts stained with anti-CD31 and MitoTracker. Three control and four mutant mice were treated with an acute dose of doxorubicin. The gates represent the cardiomyocyte population (CD31^neg^/MitoTracker^high^). (**B**) Representative histograms of the MitoTracker signal in the mutant (blue) and control (green) populations of cardiomyocytes. (**C**) Quantification of the staining shows a significant decrease in the mitochondrial load of mutant cardiomyocytes, but not in endothelial cells (means ± SD). *p* < 0.01, *Z*-test.

As it has been described that doxorubicin accumulates in mitochondria and causes mitochondrial fragmentation [22], we estimated the mitochondrial mass through MitoTracker staining and FACS in cardiac cells from mutant and control mice treated with doxorubicin. As shown in Figure 6, the median fluorescence of cardiomyocytes from mutant mice was significantly lower than the fluorescence from control cardiomyocytes.

However, there was no significant difference in the mitochondrial load of endothelial cells from mutant and control hearts.

Treatment with doxorubicin provoked a few electrocardiographic changes after three months in mutant mice as compared to controls (Table 3). These changes consisted of a shortening of the PR interval and a lengthening of the QT, JT, and Tpeak–Tend intervals. The differences between mutant and controls became statistically not significant after six months of treatment.

### 3.7. Proteomic Analysis of the Hearts from Doxorubicin-Treated Mice Shows Metabolic Alterations

Over-representation analysis (ORA) was performed on a list of 42 proteins (8 downregulated, 34 upregulated) with an abundance ratio <0.9 or >1.1, and a *p*-value < 0.05. Additionally, the whole list of 402 proteins with a high protein FDR confidence level and a minimum of two peptides identified was analyzed with a gene set enrichment analysis method (GSEA).

According to ORA analysis, biological processes significantly over-represented in the 42 proteins selected included mainly metabolic processes related to fatty acid oxidation and glycolysis/gluconeogenesis (Table 4). The main differences found were also related to fatty acid oxidation. GSEA analysis of the full set of proteins showed a downregulation of the pathways related to electron transport chain and oxidative phosphorylation, like the results found for non-treated mice. These results are not included in Table 4 since the FDR values obtained in both cases (*p* < 0.07) were statistically not significant.

Supplementary Table S5 shows 15 proteins differentially expressed in the myocardium of mutant and control mice, 4 of which were downregulated and 11 of which were upregulated in the mutant (adjusted *p*-value of <0.05). The significance of the strong upregulation of thioredoxin is discussed below.

## 4. Discussion

The prominent expression and the essential role played by WT1 in the embryonic epicardium probably obscured other functions of this transcription factor in the developing and adult heart. However, some lines of evidence have shown that WT1 is involved in the development of the myocardium. It is expressed in cardiomyocytes from at least embryonic day 10.5 until adulthood, but this expression decreases along development [6]. We previously showed that conditional deletion of WT1 in cardiac-troponin-expressing cell lineages (cardiomyocytes and a subset of epicardial cells) caused abnormal sinus venosus and atrium development, a thin ventricular myocardium, and electrocardiographic anomalies. RNA sequencing analysis suggested that both calcium ion regulation and modulation of potassium channels are deeply altered in the mutant myocardium by the ablation of WT1. In fact, three of the six most relevant biological processes altered by WT1 ablation were calcium homeostasis, regulation of its cytosolic concentration, and transport [5]. We were prompted by these results to investigate the consequences of the conditional deletion of WT1 in adult cardiomyocytes.

We performed the first study on primary cardiomyocytes isolated from newborn mice, where WT1 expression was silenced by shRNA transduction. The lower TMRE staining shown by the WT1-deficient cardiomyocytes supported mitochondrial dysfunction as TMRE fluorescence is proportional to the mitochondrial transmembrane potential. The resting intracellular calcium level (measured via Fluo4-AM staining) appeared to be slightly increased in the mutant cardiomyocytes, although the difference was statistically not significant. This observation deserves further investigation since Ca^2+^ accumulation can impair mitochondrial function, leading to reduced ATP production and increased release of reactive oxygen species [23].

Alteration in calcium homeostasis might also be related to some of the changes observed in the expression of genes from the mutant cardiomyocytes. *Stim1*, a gene downregulated after *Wt1* silencing, encodes a type 1 transmembrane protein that mediates Ca^2+^ influx after depletion of intracellular Ca^2+^ stores via the gating of store-operated Ca^2+^ influx channels. *Stim1* is regulated by WT1 [24]. Calcium/calmodulin-dependent protein kinase, also downregulated in WT1-deficient cardiomyocytes, is involved in the regulation of Ca^2+^ homeostasis in the myocardium, targeting ion channels, transporters, and accessory proteins involved in Ca^2+^ influx [25]. We consider the overexpression of the potassium channel Kcnk2 (aka Trek-1) interesting since upregulation of this protein has a protective effect against ischemia–reperfusion-induced injury and it is involved in the post-infarction cardiac remodeling via regulating membrane potential and maintaining intracellular Ca^2+^ homeostasis [26]. Thus, the changes observed after the silencing of WT1 expression in cultured cardiomyocytes suggest alterations in Ca^2+^ homeostasis after WT1 loss of function. Nevertheless, future studies are required to characterize electrophysiological properties, calcium release, and re-uptake upon silencing of WT1 in detail.

We found a significant increase in fibrosis and cardiomyocyte size in adult hearts with conditional deletion of WT1. However, a larger cardiomyocyte size was not correlated with a significant increase in the heart weight/body weight ratio, perhaps due to the higher mortality of cardiomyocytes in the mutant mice, a possibility supported by the significant increase in fibrotic tissue. Interestingly, the relative difference between mutant and control mice increased in the group of mice evaluated six months after tamoxifen administration or after Wt1 deletion, suggesting that the lack of WT1 impairs cardiac recovery. As we will discuss below, the effect of doxorubicin treatment also increased fibrosis further in WT1-deficient hearts; furthermore, again, the relative difference between mutant and controls was higher after six months of treatment. It is uncertain if this increase in fibrosis could be related to the reported shortening of the QRS interval in fibrotic hearts [27].

The relatively high level of fibrosis observed in the untreated control after two months (it is always lower than the fibrosis observed in the mutant mice) might be attributed to the tamoxifen treatment, which causes fibrosis in αMHCMerCreMer mice regardless of the floxed transgene [28]. Additionally, transient cardiomyopathy has been reported due to the expression of Cre-recombinase [29]. This effect is transitory, and it is resolved after four weeks, the time point at which we performed our study. In fact, we did not detect significant differences between the Cre+ and Cre- control mice Incidentally, the study recently published on the proteome variations provoked by Cre-recombinase expression in the MerCreMer model showed moderate changes, entirely different to those found by us in the conditional WT1-deficient mice [30]. Thus, we assume that all the features of the phenotype of αMHCMerCreMer mice are due to the downregulation of WT1 expression.

Regarding fibrosis, we detected evidence of mitochondrial dysfunction. Staining of cardiomyocytes with TMRE revealed a lower fluorescence in the mutant mice as compared with controls. The reduction (84%) was statistically significant.

This reduced mitochondrial membrane potential can be correlated with the metabolic anomalies revealed by the proteome analysis. The biological processes significantly over-represented mainly included metabolic pathways, including oxidative phosphorylation. The analysis of the full set of proteins showed a significant downregulation of the pathways related to electron transport chain and oxidative phosphorylation, which agrees with our flow cytometry results.

The analysis of individual proteins significantly upregulated in the mutant can be correlated with the results described above. Pyruvate dehydrogenase kinase 2 inhibits pyruvate dehydrogenase and increases the glycolytic pathway. Nicotinamide phosphoribosyltransferase plays an essential role in NAD^+^ biosynthesis and carbohydrate metabolism. Inhibitors of this enzyme downregulate glycolysis [31].

Serine/threonine-protein phosphatase 2A is involved in the regulation of adaptive stress responses [32], while microsomal glutathione S-transferase 3 catalyzes the oxidation of hydroxy fatty acids [33]. Glutathione S-transferase P2 has an important role in the detoxification of endogenous compounds such as peroxidized lipids. Finally, the mitochondrial methylcrotonoyl-CoA carboxylase beta chain shows ts the largest increase in expression (3-fold). This enzyme catalyzes a critical step for leucine and isovaleric acid catabolism. Thus, the changes observed in the proteome point to alterations in cardiomyocyte metabolism as well as an increase in the protection of the cells against damage.

This role played by WT1 in physiological conditions seems to become even more critical when the cardiomyocytes are under damaging conditions. Doxorubicin is an important anticancer drug, but it causes cardiotoxicity in many patients [34]. We observed that mice with myocardial deletion of WT1 and treated with doxorubicin displayed similar anomalies to control animals treated with doxorubicin, but these anomalies were aggravated and lasted longer. This is the case for interstitial fibrosis and mitochondrial dysfunction. In fact, mutant mice treated with doxorubicin showed a significant reduction in the mitochondrial load in their hearts compared with the controls, a result which was not observed in the untreated mutant mice. Furthermore, WT1-deficient mice treated with doxorubicin showed significant electrocardiographic alterations, particularly a lengthening of the QT and JT intervals indicative of cardiac repolarization defects.

Proteomic analysis of mutant hearts from doxorubicin-treated mice showed, again, upregulated proteins related to metabolic processes and cytoprotective mechanisms, for example, Enoyl-CoA hydratase and short-chain specific acyl-CoA dehydrogenase (mitochondrial enzymes involved in fatty acid beta-oxidation) and glucose-6-phosphate isomerase (glycolysis and gluconeogenesis). The strong upregulation of thioredoxin (2.6-fold in mutants) provides improved protection against oxidative stress. Additionally, the downregulation of calsequestrin-2 seems to be relevant as this protein is a key determinant of the functional size and stability of sarcoplasmic reticulum Ca^2+^ stores in cardiac muscle [35].

In summary, the whole set of results from cultured cardiomyocytes and mice with conditional deletion of WT1 in cardiomyocytes suggests that this transcription factor plays a physiological protective role in adult cardiomyocytes. This role might be mediated by regulation of Ca^2+^ homeostasis through a mechanism that remains to be determined. The role played by WT1 in the adult myocardium seems to be more important under pathophysiological conditions, and this could be related to the observation of an upregulation of WT1 expression in cardiomyocytes following myocardial infarction [6]. The activation of WT1 in pathological conditions has been already described in other cell types such as the epicardium [36], cardiac endothelium [10,11], liver [9], and pancreas [8]. Thus, knowledge of the pathways regulated by WT1 in the adult myocardium could be of great interest for the development of therapeutic strategies against cardiac pathologies [37].

## Figures and Tables

**Table 1:** ECG parameters in control mice and mice with conditional deletion of WT1 in cardiomyocytes. Only the QRS complex showed a significant decrease in mutant mice. Mean values ± SD. * *p* < 0.05.

8	RR Interval (ms)	Heart Rate (BPM)	PR Interval (ms)	P Duration (ms)	QRS Interval (ms)	QT Interval (ms)	QTc (ms)	JT Interval (ms)	Tpeak Tend Interval (ms)	P Amplitude (µV)	Q Amplitude (µV)	R Amplitude (µV)	S Amplitude (µV)	ST Height (µV)	T Amplitude (µV)
Control	249 ± 47.2	252 ± 52.0	39.1 ± 5.6	15.9 ± 2.4	14.6 ± 1.6	33.4 ± 3.5	67.9 ± 9.5	18.7 ± 3.7	12.5 ± 3.0	34.4 ± 23.6	10.9 ± 6.5	375 ± 96.5	−15.6 ± 71.6	41.9 ± 54.0	114.3 ± 49.0
	N = 31	N = 31	N = 29	N = 29	N = 31	N = 31	N = 31	N = 31	N = 31	N = 30	N = 31	N = 31	N = 31	N = 31	N = 31
Mutant	247.8 ± 46.4	250.5 ± 34.2	38.1 ± 5.6	16.6 ± 2.6	13.6 ± 1.3	32.2 ± 3.7	65.2 ± 8.3	18.4 ± 3.4	11.9 ± 2.8	34.3 ± 31.9	9.65 ± 5.3	352.5 ± 89.0	9.16 ± 43.6	57.1 ± 32.0	104.6 ± 36.1
	N = 25	N = 25	N = 25	N = 25	N = 25	N = 25	N = 25	N = 25	N = 25	N = 25	N = 25	N = 25	N = 25	N = 25	N = 25
Student’s t	0.923	0.896	0.522	0.304	0.014 *	0.227	0.270	0.718	0.408	0.982	0.448	0.371	0.136	0.221	0.409

**Table 2:** Results of proteomic analysis of hearts from four control mice and three mice with conditional deletion of WT1 in cardiomyocytes.

ORA-PATHWAY-KEGG			
Gene set description	Ratio mutant/control	*p*-value	FDR
Valine, leucine, and isoleucine degradation	7.92	<0.001	0.016
Glutathione metabolism	6.93	<0.001	0.022
Cardiac muscle contraction	6.83	<0.001	0.015
PPAR signaling pathway	6.26	<0.001	0.016
Parkinson disease	6.16	<0.00001	0.001
Oxidative phosphorylation	5.96	<0.0001	0.003
Carbon metabolism	5.92	<0.001	0.006
Ribosome	5.10	<0.001	0.013
Non-alcoholic fatty liver disease (NAFLD)	4.70	<0.001	0.015
Metabolic pathways	1.84	<0.001	0.022
			
ORA Phenotype			
Gene set description	Ratio mutant/control	*p*-value	FDR
Increased fatty acid level	8.49	<0.0001	0.028
Abnormal fatty acid level	6.61	<0.001	0.003
Abnormal circulating free fatty acids level	6.57	<0.0001	0.035
Abnormal free fatty acids level	6.26	<0.0001	0.041
Abnormal heartbeat	5.63	<0.00001	0.007
Abnormal metabolism	3.72	<0.001	0.003
			
GSEA analysis pathway according to WikiPathways			
Gene set description	NES mutant/control	*p*-value	FDR
Electron transport chain	−2.68	<0.0001	<0.0001
Oxidative phosphorylation	−3.26	<0.0001	<0.0001

Only results with an FDR < 0.05 are represented. The main pathways altered by the deletion correspond to metabolic processes. NES: normalized enrichment score.

**Table 3:** ECG parameters in control mice and mice with conditional deletion of WT1 in cardiomyocytes after treatment with doxorubicin. A significant lengthening of the JT and Tpeak–Tend intervals was observed three months after the treatment, although these alterations diminished after six months. Increased QT and QTc and decreased PR intervals were also observed in mutant mice. Mean values ± SD. * *p* < 0.05, ** *p* < 0.01.

		RR Interval (ms)	Heart Rate (BPM)	PR Interval (ms)	P Duration (ms)	QRS Interval (ms)	QT Interval (ms)	QTc (ms)	JT Interval (ms)	Tpeak Tend Interval (ms)	P Amplitude (µV)	Q Amplitude (µV)	R Amplitude (µV)	S Amplitude (µV)	ST Height (µV)	T Amplitude (µV)
	Control	253.3 ± 19.6	238.6 ± 17.9	40.5 ± 1.8	15.0 ± 1.0	13.7 ± 1.3	29.0 ± 4.4	57.7 ± 9.5	14.2 ± 2.6	8.7 ± 2.1	43.0 ± 12.5	12.2 ± 2.8	314.9 ± 88.2	5.29 ± 82.9	48.6 ± 33.3	72.6 ± 25.3
Three months		N = 9	N = 9	N = 8	N = 8	N = 9	N = 9	N = 9	N = 9	N = 9	N = 9	N = 9	N = 9	N = 9	N = 9	N = 9
	Mutant	269.2 ± 35.2	232.5 ± 229.0	36.1 ± 5.7	14.7 ± 2.2	13.5 ± 1.2	33.2 ± 4.7	64.8 ± 6.5	19.8 ± 4.3	13.6 ± 3.9	20.7 ± 36.4	12.3 ± 4.0	300.6 ± 71.6	5.4 ± 53.8	74.1 ± 34.5	122.7 ± 44.0
		N = 6	N = 6	N = 6	N = 6	N = 6	N = 6	N = 6	N = 6	N = 6	N = 6	N = 6	N = 6	N = 6	N = 6	N = 6
	Student’s t	0.280	0.562	0.059	0.688	0.711	0.102	0.133	0.008 **	0.007 **	0.110	0.968	0.747	0.786	0.174	0.015 *
	Control	232.9 ± 45.7	267.4 ± 52.9	37.8 ± 4.1	14.5 ± 2.8	13.3 ± 1.1	29.7 ± 5.6	63.7 ± 15.0	13.8 ± 4.5	8.67 ± 3.4	25.6 ± 3.0	12.2 ± 4.3	331.0 ± 103.1	20.2 ± 88.4	55.1 ± 35.0	58.2 ± 50.0
Six months		N = 9	N = 9	N = 8	N = 8	N = 9	N = 8	N = 8	N = 8	N = 8	N = 8	N = 9	N = 9	N = 9	N = 9	N = 9
	Mutant	256.9 ± 4.5	240.4 ± 43.7	38.8 ± 4.6	14.6 ± 0.95	14.0 ± 1.7	30.9 ± 3.9	61.7 ± 10.8	16.9 ± 3.0	11.0 ± 2.5	48.9 ± 34.0	10.0 ± 4.4	307.9 ± 68.2	−17.4 ± 63.2	55.5 ± 42.8	114.5 ± 35.8
		N = 6	N = 6	N = 5	N = 5	N = 6	N = 6	N = 6	N = 6	N = 6	N = 6	N = 6	N = 6	N = 6	N = 6	N = 6
	Student’s t	0.883	0.179	0.790	0.787	0.852	0.854	0.8589	0.852	0.851	0.622	0.226	0.175	0.942	0.322	0.538

**Table 4:** Results of proteomic analysis of hearts from two control mice and three mice with conditional deletion of WT1 in cardiomyocytes after chronic doxorubicin treatment. Only results with FDR < 0.05 are represented. The pathways altered by the deletion correspond to fatty acid oxidation and glycolysis.

ORA-PATHWAY-WIKI			
Gene set description	Ratio mutant/control	*p*-value	FDR
Fatty acid oxidation	54.18	<0.001	0.035
Fatty acid beta oxidation	23.90	<0.001	0.021
Glycolysis and gluconeogenesis	21.25	<0.0001	0.005

## Data Availability

Supporting data can be found in the RIUMA (Institutional Repository of the University of Málaga).

## Supplementary Materials

The following supporting information can be downloaded at: https://www.mdpi.com/2308-3425/10/5/211/s1. Table S1: Number of mice used for each Experiment; Table S2: Primers used for qRT-PCR; Table S3: Antibodies used for Western blot, flow cytometry and immunofluorescence; Table S4: Proteins differentially expressed in the heart of control mice and mice with conditional deletion of WT1 in cardiomyocytes; Table S5: Proteins differentially expressed in the heart of control mice and mice with conditional deletion of WT1 in cardiomyocytes after chronic doxorubicin treatment.
