# Correction: Díaz del Moral et al. Cardiomyocyte-Specific Wt1 Is Involved in Cardiac Metabolism and Response to Damage. *J. Cardiovasc. Dev. Dis.* 2023, *10*, 211

**DOI:** 10.3390/jcdd11070190

**Published:** 2024-06-24

**Authors:** Sandra Díaz del Moral, Maha Benaouicha, Cristina Villa del Campo, Miguel Torres, Nicole Wagner, Kay-Dietrich Wagner, Ramón Muñoz-Chápuli, Rita Carmona

**Affiliations:** 1 Department of Animal Biology, Faculty of Science, University of Málaga, 29071 Málaga, Spain; sandradiaz@uma.es (S.D.d.M.); chapuli@uma.es (R.M.-C.); 2 Department of Cell Biology, Genetics and Physiology, Faculty of Science, University of Málaga, 29071 Málaga, Spain; benaouicha.maha@gmail.com; 3 Cardiovascular Development Program, Centro Nacional de Investigaciones Cardiovasculares, CNIC, 28029 Madrid, Spain; cristina.villa@cnic.es (C.V.d.C.); mtorres@cnic.es (M.T.); 4 Centro de Investigación Biomédica en Red de Enfermedades Cardiovasculares (CIBERCV), 28029 Madrid, Spain; 5 Université Côte d’Azur, CNRS, INSERM, iBV, 06108 Nice, France; nwagner@unice.fr (N.W.); kwagner@unice.fr (K.-D.W.); 6 Department of Human Anatomy, Legal Medicine and History of Science, Faculty of Medicine, University of Málaga, 29071 Málaga, Spain

In the published publication [1], there was an error regarding the affiliation for Cristina Villa del Campo and Miguel Torres. In addition to affiliation 3, the updated affiliation should include affiliation 4: Centro de Investigación Biomédica en Red de Enfermedades Cardiovasculares (CIBERCV), 28029 Madrid, Spain. The authors state that the scientific conclusions are unaffected. This correction was approved by the Academic Editor. The original publication was also updated.
