# Supplementary material for: Cardiomyocyte-Specific Wt1 Is Involved in Cardiac Metabolism and Response to Damage
Source: J Cardiovasc Dev Dis. 2023 May 12;10(5):211. doi: 10.3390/jcdd10050211 (PMC10219250; doi:10.3390/jcdd10050211)
Supplement: Supplementary file 1 [file jcdd-10-00211-s001.zip › jcdd-2347826-supplementary.pdf]

Supplementary Table S1. Number of mice used for each Experiment. A total number of 31 control and 25 mutant mice were included in the study. ECGs for all these animals are shown in Table 1. 22 mice (12 controls, 10 mutants) were treated with doxorubicin, either chronically or acutely. Seven mice died after the tamoxifen treatment and before the experiments, without significant differences between control and mutants. Some untreated mice were unsuccessfully employed for cardiomyocyte isolation through FACS (not included in the results). Some samples from the same mice were used for two different techniques.

			ECG before Doxorubicin	ECG after Doxorubicin	Fibrosis (2/6 months)	Hypertrophy	Oxidative stress	Cytometry	Proteomics
Untreated		Control	19		4	2	3	4	4
		Mutant	15		5	3	3	7	3
Treated with Doxorubicin	Chronic	Control	9	9	3		3		2
		Mutant	6	6	3		3		3
	Acute	Control	3		3			3	
		Mutant	4		4			4	

Supplementary Table S2. Primers used for qRT-PCR.

Gene	Oligonucleotide Sequences
Gapdh	F: AGGTCGGTGTGAACGGATTTG<br>R: TGTAGACCATGTAGTTGAGGTCA
β-actin	F: CTTCCTCCCTGGAGAAGAGC<br>R: ATGCCACAGGATTCCATACC
Rplp0	F: CACTGGTCTAGGACCCGAGAAG<br>R: GGTGCCTCTGGAGATTTTCG
Wt1	F: GCCTTCACCTTGCACTTCTC<br>R: CGAAAGTGACCGTGCTGTAT
Stim1	F: CTGGGATCTCAGAGGGATTTGA<br>R: AGGCATGGCATTGAGAGCTT
Tcf4	F: AAGAAAGTCCAAAAGTTCC<br>R: CATCTTGCATGAAGAAGGAG
Tead2	F: GATAGAGTTCTCAGCGTTTG<br>R: TTCTTCTCAGGGAATTTGTC
CamkIId	F: GCTTTCTGCTAGGGACCATCA<br>R: ACTGGCATCAGCTTCACTGT
Kcnk2	F: CCATAGGATTTGGAAACATCTC<br>R: AAATGTGTCTTCCACTTTGG
Cacng7	F: GAATATTCTGAAGACAGTGCG<br>R: TTGATGCTGGAGATGTATAGG
Kcna5	F: ATTGGTGCTTTGTGTCTAAG<br>R: ATGCTTCTGTGAAGAAAGTC
Tnnt2	F: TTCGACCTGCAGGAAAAGTT<br>R: GCACAGCTTTGACGAGAACA

Supplementary Table S3. Antibodies used for Western blot, flow cytometry and immunofluorescence.

Antibody	Supplier	Reference	Dilution
Rabbit monoclonal anti-WT1	Abcam	Ab89901	1/1000
Rabbit monoclonal anti-Gapdh	Abcam	Ab181602	1/1000
Rabbit polyclonal anti-Laminin	Sigma	L9393	1/25
Mouse monoclonal anti-CD31 APC	Thermo scientific	17-0311-82	1/100
Mouse monoclonal anti-CD31 PE	Thermo scientific	12-0311-82	1/100

Supplementary Table S4. Proteins differentially expressed in the heart of control mice and mice with conditional deletion of WT1 in cardiomyocytes. Only proteins with an adjusted p-value of <0.05 are shown.

**Protein**	**Abundance Ratio: (KO/WT)**	**Abundance Ratio p-value: (KO/WT)**	**Abundance Ratio Adj. P-value: (KO/WT)**
40S ribosomal protein S28 [OS=Mus musculus]	0.76	1.8068E-05	0.00543673
Hemopexin [OS=Mus musculus]	0.786	0.00100825	0.04756075
Bcl-2-like protein 13 [OS=Mus musculus]	0.839	0.00104358	0.04785978
Plasminogen activator inhibitor 1 RNA-binding protein [OS=Mus musculus]	1.108	0.00013922	0.0191548
[Pyruvate dehydrogenase (acetyl-transferring)] kinase isozyme 2, mitochondrial [OS=Mus musculus]	1.149	0.00010955	0.0191548
Microsomal glutathione S-transferase 3 [OS=Mus musculus]	1.184	0.00015607	0.01982146
Methylcrotonoyl-CoA carboxylase beta chain, mitochondrial [OS=Mus musculus]	3.103	0.00042616	0.03517967
Nicotinamide phosphoribosyltransferase [OS=Mus musculus]	1.125	0.00051385	0.03637986
Nucleosome assembly protein 1-like 4 [OS=Mus musculus]	1.163	0.00057641	0.03637986
Serine/threonine-protein phosphatase 2A 65 kDa regulatory subunit A alpha isoform [OS=Mus musculus]	1.224	0.00048514	0.03637986
T-complex protein 1 subunit delta [OS=Mus musculus]	1.342	0.00048022	0.03637986
Very-long-chain (3R)-3-hydroxyacyl-CoA dehydratase 2 [OS=Mus musculus]	1.418	0.00074713	0.03979073
Glutathione S-transferase P 2 [OS=Mus musculus]	1.19	0.00086769	0.04213413

Supplementary Table S5. Proteins differentially expressed in the heart of control mice and mice with conditional deletion of WT1 in cardiomyocytes after chronic doxorubicin treatment. Only proteins with an adjusted p-value of <0.05 are shown.

Protein	Abundance Ratio: (KO/WT)	Abundance Ratio p-value: (KO/WT)	Abundance Ratio Adj. P-value: (KO/WT)
myosin-6 [OS=Mus musculus]	0.896	0.00056	0.0333711
Long-chain-fatty-acid--CoA ligase 1 [OS=Mus musculus]	0.913	5.21E-05	0.00778969
Calsequestrin-2 [OS=Mus musculus]	0.93	0.00046	0.03328712
Ras-related protein Rap-1b [OS=Mus musculus]	0.95	1.55E-05	0.00386072
Enoyl-CoA hydratase, mitochondrial [OS=Mus musculus]	1.056	0.00059	0.0333711
Prelamin-A/C [OS=Mus musculus]	1.088	0.00104	0.0333711
ubiquitin-conjugating enzyme E2 N [OS=Mus musculus]	1.09	0.00043	0.03328712
glucose-6-phosphate isomerase [OS=Mus musculus]	1.122	0.00154	0.04127835
Short-chain specific acyl-CoA dehydrogenase, mitochondrial [OS=Mus musculus]	1.133	0.00028	0.02634984
Acylphosphatase-2 [OS=Mus musculus]	1.191	0.00049	0.03328712
Histone H2B type 1-B [OS=Mus musculus]	1.219	0.0011165	0.0333711
peptidyl-prolyl cis-trans isomerase A [OS=Mus musculus]	1.23	3.53E-05	0.00660217
Aspartate aminotransferase, cytoplasmic [OS=Mus musculus]	1.241	0.00014	0.01847603
Translationally-controlled tumor protein [OS=Mus musculus]	1.246	0.00168	0.0434786
thioredoxin [OS=Mus musculus]	2.596	0.00132	0.03810134
